# Upcycled vs. Sustainable: Identifying Consumer Segments and Recognition of Sustainable and Upcycled Foods Within the United States

**DOI:** 10.3390/foods14203508

**Published:** 2025-10-15

**Authors:** Karissa Chu, Daniel DeGeorge, Dan Diehn, Alissa Galatz, Jeff Garza, Lucy McGowan, MaryAnne Drake, Samir Amin, Amy Lammert

**Affiliations:** 1 Food Science & Nutrition Department, California Polytechnic State University, San Luis Obispo, CA 93407, USA; kchu23@calpoly.edu (K.C.); djdegeor@calpoly.edu (D.D.); samin02@calpoly.edu (S.A.); 2 Garza Consulting LLC, Grand Rapids, MI 49525, USA; dan.diehn@the-gc.com (D.D.); alissa.galatz@the-gc.com (A.G.); jeff.garza@the-gc.com (J.G.); 3 Department of Agribusiness, California Polytechnic State University, San Luis Obispo, CA 93407, USA; lumcgowa@calpoly.edu; 4 Department of Food, Bioprocessing and Nutrition Sciences, North Carolina State University, Raleigh, NC 27606, USA; mdrake@ncsu.edu

**Keywords:** upcycled, sustainable, consumer segmentation, survey

## Abstract

Upcycled foods are a rising trend as the issue of food waste and sustainability becomes an increasing concern. The objective of this research was to evaluate the perception of upcycled foods through the characterization of consumer segments. An online questionnaire was developed to evaluate food neophobia, lifestyle, behavior, beliefs, awareness, and familiarity or recognition of upcycled and sustainable food products using a pre- and post-infographic intervention. The survey was created using Red Jade SaaS and distributed to participants through the Cal Poly Sensory database, North Carolina State University Sensory Service Center database, social media (LinkedIn and Nextdoor), and personal communication. Participants (*n* = 947) were segmented using a k-means clustering algorithm on lifestyle, neophobia, and beliefs questions. Four clusters were identified: Greenthusiasts (*n* = 306)—environmentally conscious and open-minded to new products, Balanced Believers (*n* = 347)—supportive of new products with reasonable doubt, Healthy Hesitants (*n* = 208)—willing to make health-focused changes but hesitant towards new products and technologies, and Eco-Skeptics (*n* = 86)—doubtful and resistant to change, most food and technologically neophobic. Based on pre- and post-intervention, Eco-Skeptics had the lowest initial awareness and recognition of sustainable and upcycled food products, while Greenthusiasts had the highest. All four segments trended towards improved recognition of the food products post-intervention.

## 1. Introduction

With the growing human population, estimated to reach 9 billion by the year 2050 [1], there is a need for a more sustainable food system to satisfy this food demand. Sustainability can be viewed as an overarching term that encompasses conserving water, land, energy, and natural resources. The Food Waste Index Report [2] estimates that in 2022, approximately 1.05 billion tons of food waste were generated. Waste is commonly produced through a surplus of food produced or byproducts that are not used in the final product. This loss translates to wasted water, land, energy, and natural resources that devastate the economy and the environment [3]. Upcycled foods are a promising solution to divert the generated food waste from the landfill as they incorporate ingredients that previously would not have been consumed [4], therefore, addressing significant sustainability issues.

There has already been an increase in products on the market and research on upcycled foods, which are well summarized by Lu et al. [5], as well as Yilmaz and Kahveci [6]. This research has observed a trend of the younger population showing a higher interest in upcycled foods, particularly those between 18 and 34 years old [7], while those in the older population express less interest [6,8,9]. However, according to Zhang et al. [10], Gen Z (18–27), Gen Y (Millennials, 28–43), and Baby Boomers (60–78) are more likely to purchase upcycled foods, while Gen X (44–59) are concerned about the quality, causing them to have lower intentions to purchase. The effect of gender has had mixed conclusions, where some studies suggest females have more positive attitudes and a higher purchase intent [6,7,9], some assert it depends on the product and messaging [11,12,13,14], while others have found no impact [15,16,17]. It is also currently debatable how education level or household income has an impact on the acceptance of upcycled foods. Coderoni and Perito [12,16] found that college students and those with a lower income were more likely to have a more positive attitude or were more willing to purchase upcycled foods. Although Cattaneo et al. [18] and Perito et al. [19] agreed that higher education leads to a more positive attitude towards upcycled products, alternatively, Ali et al. [15] found that a higher education led to lower purchase intent, and Yang et al. [14] concluded that education has no effect. Similarly, though Yilmaz and Kahveci [6] agreed with the income factor, McCarthy et al. [9] stated that those with a higher income found upcycled products more appealing. Employment was found not to be a significant factor, though Perito et al. [19] mentioned that students were more likely to try products containing upcycled olive leaf, and McCarthy et al. [9] noted a correlation between employment status and other demographics, which did influence participants’ willingness to purchase. Few articles have investigated the significance of ethnic background or country of origin. However, Grasso et al. [20] found that participants in the U.S. demonstrated a higher liking towards upcycled foods than those in China. Additionally, Murillo et al. [13] discovered that those with Hispanic/Latin/Spanish and Black/African American ethnic backgrounds were more willing to try foods containing seafood byproducts.

Outside of the United States (U.S.), namely, Europe, upcycled foods have been promoted through policy. Sustainability goals have been implemented by law with programs like the Green Deal and the Farm-to-Fork Strategy, specifically focusing on food waste reduction and educational initiatives [21], which can help drive the success of upcycled foods. Europe does have strict regulations on upcycled food products and claims, with many having to undergo evaluation as determined by the Novel Food Regulation [22]. Within the U.S., goals have been jointly established by the United States Department of Agriculture, Environmental Protection Agency, and Food and Drug Administration to reduce food waste [23], but there is no law that directly targets the issue of food waste, which leads to a reliance on the food industry to solve this problem. Contrary to Europe, the U.S. appears to be more lenient regarding upcycled food regulation, as many upcycled ingredients fall under the pre-existing Generally Recognized as Safe status [22]. The primary hurdle for upcycled products in the U.S. is upcycled certification, which is a private voluntary label with standards developed by the incorporation, Where Food Comes From [24]. As a result of these differences in consumer culture, opinions on upcycled foods may vary by region, which is an important consideration in determining acceptance.

Personal opinions have a major influence on purchasing and consumption behavior, which have been shown to provide insight into how they may react to upcycled foods. Food and food technology neophobia have been commonly investigated, especially with novel product types, using the Food Neophobia Scale (FNS) developed by Pliner and Hobden [25], and the Food Technology Neophobia Scale (FTNS) developed by Cox and Evans [26]. Research has provided strong evidence that those with higher neophobia are more likely to perceive upcycled foods negatively [7,12,19,27,28,29]. Other behavioral factors include taste, frugality, critical thinking, food purchasing, social desirability, and health or environmental considerations. Proserpio et al. [17] found that consumers who crave sweets and use food as a reward had higher expectations for upcycled foods. Meanwhile, Aschemann-Witzel et al. [7] found that consumers who appear to be more frugal were likely to favor upcycled bakery, dairy, and snack foods. Yang et al. [14] found that participants who were mentally stimulated with the ability to think about examples and benefits of food products had an increased purchase intent for upcycled foods. Food characteristics and purchasing factors have also appeared to be influential, where preference for upcycled foods was dependent on product category [20,30], taste [17], quality [6,31,32], origin of upcycled ingredients [7,12,15,19,33], price [6,8,31,32,34], convenience [9,28], brand [11,12], labels, logos, and certifications [8,12,15,16,30,31,32,35,36,37]. Social status has been determined to have an influence on consumers who purchase products for the following reasons: their friends do so, it is morally correct, they are loyal to brands, they think that name brands bring them prestige and distinguish them, they avoid generally bought items, and they try to buy new foods first [9,12,27,38]. The influence of health and environmental considerations has also been investigated, including natural or organic products, sustainable products, the environmental impact of products, understanding whether foods consumed are nutritious or healthy, attempting to modify diets to be healthier, concern for food waste, exercise, and trust in strangers or the food industry [9,12,13,17,38].

Current awareness of upcycled foods is low among consumers. Grasso et al. [20] found that only 20% of consumers surveyed in the U.S. have heard of upcycled foods. However, research has shown that presenting consumers with information about upcycled foods and their environmental benefits may increase acceptability [16,38,39,40]. This implies that it is vital to understand proper education methods to increase exposure to upcycled foods among consumers. Additionally, because of the novelty of upcycled products, it is important for consumers to be able to distinguish them from other sustainable products, such as plant-based alternatives or environmentally friendly ingredients.

A methodology of segmenting consumer populations was employed by Rovai et al. [41], which aimed to identify early adopters of edible insects. Edible insects are a much more novel type of product, but the emerging trend of upcycled foods indicates the potential for a similar segmentation to be identified. By adopting a similar methodology, receptive consumers of upcycled foods can be identified, which, in turn, increases the opportunity for targeted marketing strategies, the development of products, and overall exposure. This research aims to provide a comprehensive characterization of consumer behavior, attitudes, and knowledge on sustainable and upcycled foods to evaluate the impact on their perception of upcycled foods.

## 2. Materials and Methods

**Figure 1:**
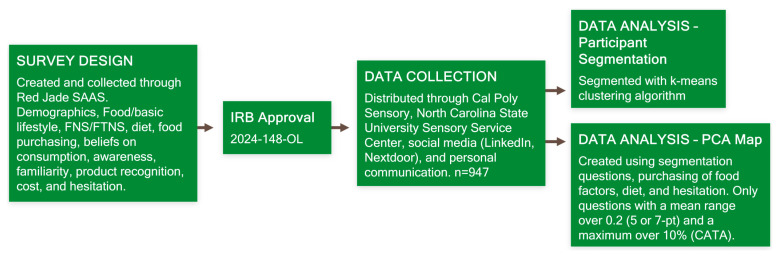
Flowchart of full study methodology.

Figure 1 summarizes the complete methodology of this study from questionnaire development to data analysis.

### 2.1. Questionnaire Design and Data Collection

**Figure 2:**
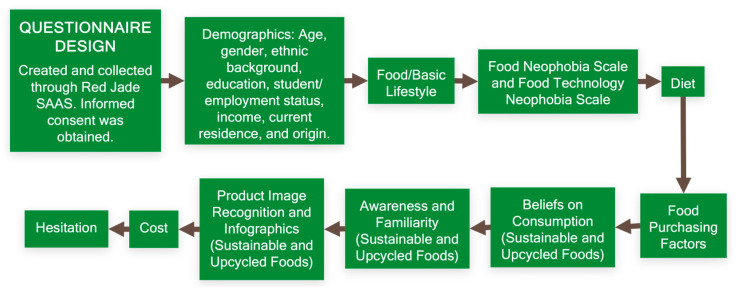
Flowchart of questionnaire design. See Supplementary Materials Document S1 for full survey questions.

An online questionnaire (Figure 1: Survey Design, Figure 2) was programmed using Red Jade SaaS (version 7.6.0) to identify participants’ lifestyles, behavior, neophobia, awareness and familiarity with sustainable and upcycled foods, and recognition of sustainable and upcycled foods. Participants completed this questionnaire online through the Red Jade platform. They were recruited via email from the Cal Poly Sensory database, the North Carolina State University Sensory Service Center database, LinkedIn, Nextdoor, and personal communication (Figure 1: Data Collection). The exact number of respondents from each of these sources is unknown.

The questionnaire (Supplementary Materials Document S1) was designed to (a) characterize segments of participants based on their lifestyles, neophobia, and beliefs about the consumption of sustainable and upcycled foods, (b) identify segments which indicate a higher likelihood of acceptance towards sustainable and upcycled foods, (c) compare segments based on demographics, diet, purchasing of food factors, cost, and hesitation towards consuming or purchasing sustainable and upcycled foods, and (d) determine the influence of informing participants of sustainable and upcycled foods on their recognition.

#### 2.1.1. Demographics

Demographics were gathered, including age, gender, ethnic background, education level, student or employment status, household income, and current residence/origin (by U.S. region). Origin was included to evaluate if there was an impact of where a participant was originally from versus where they were currently residing on their responses.

#### 2.1.2. Food and Basic Lifestyle

Food and basic lifestyle made up a total of 25 questions of the complete survey. Thirteen of these were food lifestyle questions on a 5-point Likert scale (1 = Strongly Disagree to 5 = Strongly Agree), which were presented in a randomized order. The following 12 questions were basic lifestyle questions, also on a 5-point Likert scale (1 = Strongly Disagree to 5 = Strongly Agree), and were presented in a randomized order. These questions were chosen because they have been shown to be influential in characterizing those with stronger opinions on upcycled foods.

#### 2.1.3. Food Neophobia and Food Technology Neophobia Scales

Due to the novelty of some upcycled products on the market and technologies used to develop them, participants were prompted with the FNS [25] and the FTNS [26], both on a 7-point Likert scale (1 = Strongly Disagree to 7 = Strongly Agree).

#### 2.1.4. Diet and Food Purchasing Factors

Dietary restrictions were evaluated through check-all-that-apply (CATA) questions, adopted from Rovai et al. [41], and presented in a randomized order (eat almost anything; healthy eating, but no particular nutritional restrictions; on again, off again healthy eating; flexitarian; clean; ketogenic; paleo; whole 30/anti-inflammatory; pescatarian; vegetarian; vegan; gluten-free; dairy-free; intermittent fasting; and low-glycemic). A 5-point Likert scale (1 = Not Important at all to 5 = Very Important) was used for the purchasing of food factors (cost, convenience, nutritional value, upcycled content, origin, brand, label, logo, certification, taste, quality, product category, locality, sustainability, social impact, and traceability). Factors were presented in a random order to participants. These questions were included to determine the importance of health and general considerations in food choices.

#### 2.1.5. Influence of Beliefs on Consumption

Twelve questions were asked regarding consumer beliefs on consuming sustainable and upcycled foods, using a 5-point Likert scale (1 = Strongly Disagree to 5 = Strongly Agree). The first four were self-beliefs on consuming sustainable foods, with descriptors from Smith and Gregory [42] and Grygorczyk and Blake [43]. The following questions were self-beliefs on consuming upcycled foods, with descriptors from Aschemann-Witzel et al. [44] and Moshtaghian et al. [45]. These questions represent various elements that make up what sustainable and upcycled foods are, and they were asked in a randomized order. The last four questions were regarding the participants’ beliefs about others consuming sustainable and upcycled foods, and they were also asked in a randomized order. These questions were chosen as they indicate participants’ acceptance of these foods.

#### 2.1.6. Awareness and Familiarity

Awareness of sustainable and upcycled foods was evaluated on a 3-point scale (No, I have never heard of it; Yes, I have heard of it but I don’t know what it means; Yes, I have heard of it and I know what it means). Level of familiarity with these foods followed each of the two “Yes” responses (I am familiar with the concept of sustainability/upcycling, I know what sustainable/upcycled food is, I can think of examples of sustainable/upcycled foods, I have eaten sustainable/upcycled foods, I have purchased sustainable/upcycled foods, I have read about and/or seen videos/documentaries about sustainable/upcycled foods, I have heard about sustainable/upcycled foods through marketing products and/or word of mouth). Familiarity with sustainable and upcycled foods was also assessed using questions on a 3-point scale (No, Maybe, Yes) in a randomized order within their respective food type. These questions were adopted and modified from Rovai et al. [41] to determine how consumers are accessing information about upcycled foods.

#### 2.1.7. Product Recognition and Role of Information

**Figure 3:**
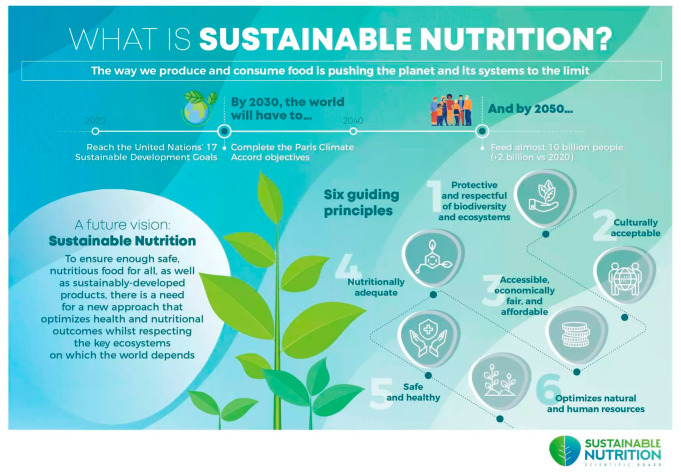
What is Sustainable Nutrition? Infographic taken from www.sustainablenutrition-sb.com and developed by the Sustainable Nutrition Scientific Board [64].

**Figure 4:**
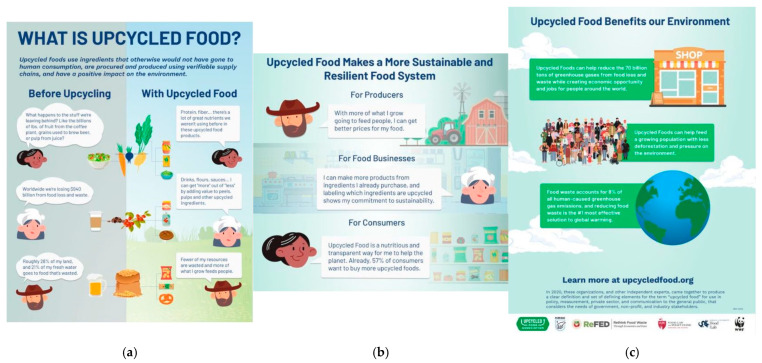
What is Upcycled Food? Infographic created by the Upcycled Food Association [65]. The original infographic was split to preserve image quality: (**a**) Top panel of the infographic, (**b**) middle panel of the infographic, (**c**) bottom panel of the infographic.

Questions linked to eleven product images (Table 1), involving five upcycled products, five sustainable products, and one attention check (neither), were presented to participants in a randomized order, who were then asked to identify if each product was sustainable, upcycled, or neither. Five food categories were used to determine product types: meat, bakery products, grain-based products, dairy-based products, and ingredient foods. As these categories have been implemented in prior research involving upcycled food, they were adopted into this study as a method of evaluating recognition of products on the market [30]. The upcycled product images (Table 1) included Flock Foods chicken skin crisps [46,47], Fancypants Baking Co. cookies [48,49], Regrained granola bars [50,51], Ascent whey protein [52], and Matriark Foods tomato sauce [53,54]. The sustainable products (Table 1) included Impossible Foods ground beef [55], Rule Breaker cookies [56,57], Grain Berry cereals [58], Brave Robot ice cream [59,60], and Revol Greens lettuce [61,62]. Due to the length of the survey, an attention check question was implemented, showing participants a photo of avocados and bananas [63], and asking them to select the “Neither” option. This question was randomized within the set of product images. After respondents viewed the eleven images and answered their respective recognition questions, they were shown infographics (Figure 3 and Figure 4) that defined what sustainable and upcycled foods are and were then asked to answer the eleven identification questions again. The sustainability infographic (Figure 3) was obtained from the Sustainable Nutrition Scientific Board [64], while the upcycled infographic (Figure 4) was from the Upcycled Food Association [65]. In order to preserve the image quality, the upcycled food infographic was split into three separate photos. Product recognition questions were included to evaluate if consumers are able to differentiate upcycled foods from other types of sustainable products (plant-based alternatives and environmentally friendly crops or ingredients) and how educating them on these concepts influences their knowledge and recognition.

#### 2.1.8. Cost and Hesitation

Participants’ willingness to pay for upcycled foods were evaluated on a 4-point scale (1 = I would be willing to pay slightly more for an upcycled product; 2 = I would buy an upcycled product if the price was similar to the traditional product; 3 = I would buy an upcycled product if the price was slightly less than the traditional product; and 4 = I would NOT buy an upcycled product, regardless of price).

A CATA question was also used in assessing hesitation towards consuming or purchasing upcycled foods. Prompts were asked in a randomized order: I think that upcycled foods are disgusting; I have a feeling that upcycled foods would not taste good; I think that upcycled foods seem strange/unfamiliar; I think that upcycled foods would be more expensive than conventional foods; They are waste products and I would not like to have them in new foods; I am not interested in their health benefits; I am not interested in their environmental benefits; I am NOT hesitant to eat upcycled foods (fixed position); and Other (fixed position) [8,66]. These questions were asked at the end of the survey to evaluate consumer opinions after learning about sustainable and upcycled foods. As an additional attention check, a prompt was included in the randomized list of hesitation questions (Do not select this).

### 2.2. Analysis

#### 2.2.1. Participants

Data from participants were analyzed for those over the age of 18 who completed the entire questionnaire. In total, 1168 questionnaires were completed, and 947 participants were included in the final segmentation and data analysis due to the elimination of those who incorrectly answered attention checks and flat responders. A total of 195 participants failed the first attention check, 16 failed the second, and 2 failed the third attention check. Data from five participants who resided out of the country were also excluded due to the proportion of those responses being marginal.

#### 2.2.2. Cluster Segmentation

Based on their responses to 60 questions throughout the survey, participants were segmented. These 60 questions included 25 food and basic lifestyle questions, 10 FNS questions, 13 FTNS questions, and 12 questions about self-belief and the belief of others regarding consumption. All of these questions were included in the segmentation because of their capability to differentiate and characterize segments within the population for those who may be more likely to accept sustainable and upcycled foods.

The questions used to segment responses were standardized within the 5-point scale and the 7-point scale for each person. Segments were determined using a k-means clustering algorithm in R (Version 4.1.3, R Studio 2023.12.0). Four clusters were identified and used for further analysis (*n* = 947). Aside from participants outside of the country and those who incorrectly answered attention checks, three participants were excluded from cluster analysis due to zero variability across the segmentation questions.

#### 2.2.3. Segment Comparison

Using scaled cluster response means, weighted study population means, and CATA percentages, a principal component analysis (PCA) was performed to understand the differences between clusters. Data for the map included the 25 food and basic lifestyle questions (5-point Likert scale), 23 FNS and FTNS questions (7-point Likert scale), 12 beliefs on consumption questions (5-point Likert scale), diet (CATA), food purchasing factors (5-point Likert scale), and hesitation questions (CATA). Responses where the range of means across clusters was less than 0.2 and the CATA response rates were less than 10% were excluded in the completion of the PCA map. These thresholds were defined to increase clarity of the maps, removing questions that were less meaningful in characterizing each segment.

As segmentation removed the assumption of randomization in the data, significance testing was not performed. To analyze demographics, sustainability and upcycling awareness, sustainable and upcycled food familiarity, product recognition, and cost of upcycled foods, segments were compared based on response percentages.

### 2.3. Ethics Statement

This research was reviewed and approved by the Cal Poly San Luis Obispo Institutional Review Board (2024-148-OL).

## 3. Results

### 3.1. Cluster Segmentation

Four segments of consumers were identified using cluster analysis. To distinguish between the four, the following names were assigned:

Cluster 1 (*n* = 306) = Greenthusiasts;
Cluster 2 (*n* = 347) = Balanced Believers;
Cluster 3 (*n* = 208) = Healthy Hesitants;
Cluster 4 (*n* = 86) = Eco-Skeptics.

**Figure 5:**
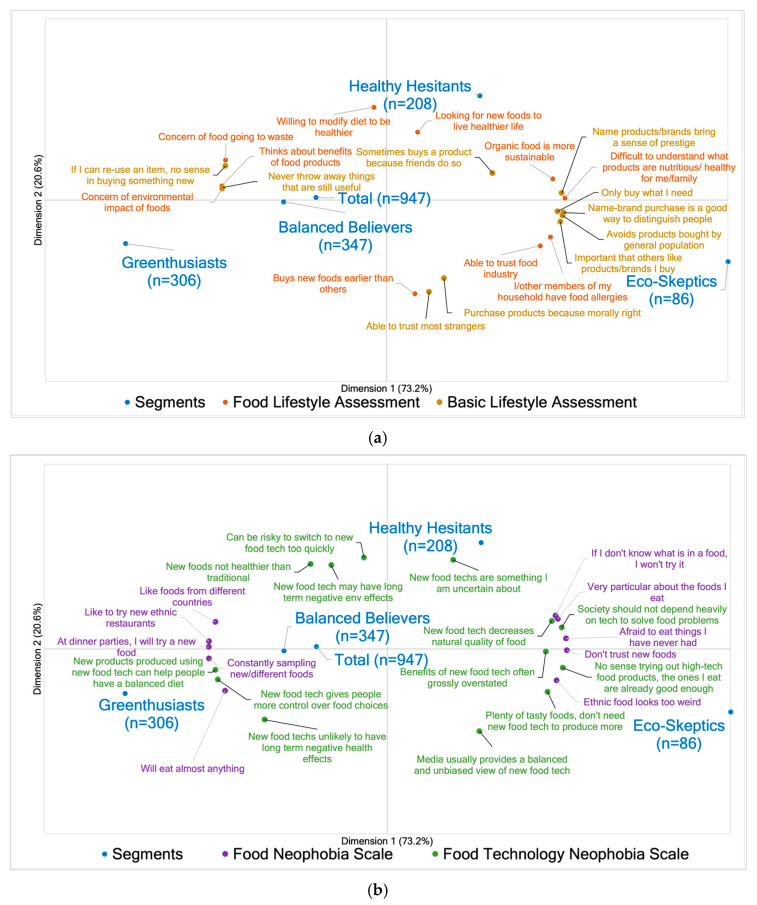

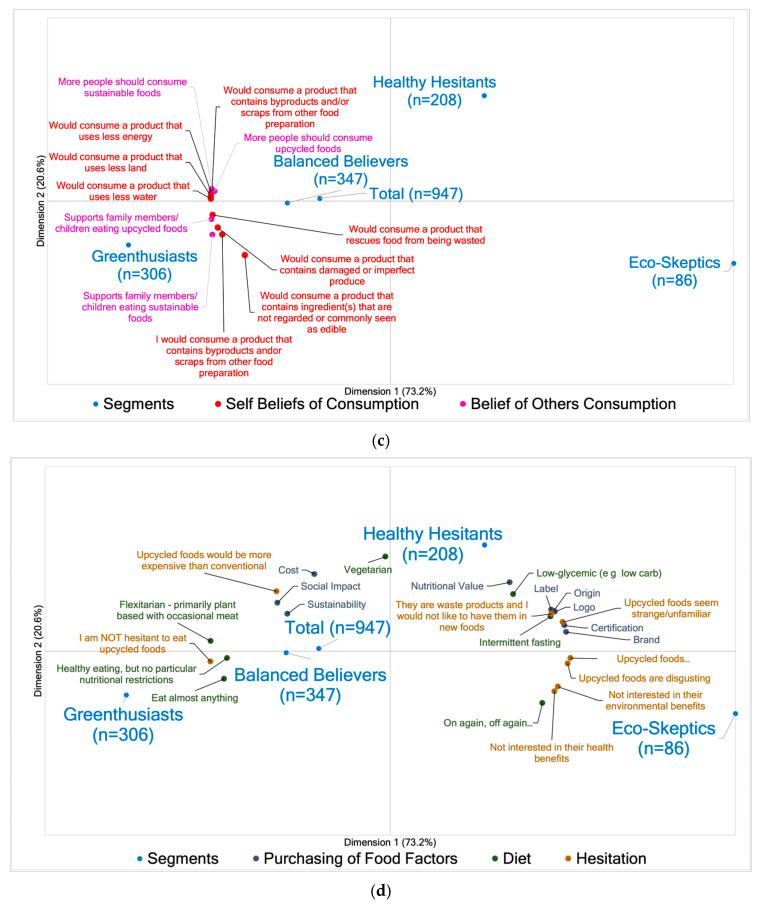
Principal component analysis (PCA) maps of segmentation, food purchasing factors, diet, and hesitation questions with the four clusters. Questions from (**a**–**d**) were jointly used to create one PCA map. These were divided for ease of viewing: (**a**) a PCA map of food and basic lifestyle assessment with the four clusters, (**b**) a PCA map of Food and Food Technology Neophobia Scales with the four clusters, (**c**) a PCA map of self beliefs and belief of others on consumption with the four clusters, (**d**) a PCA map of purchasing of food factors, diet, and hesitation with the four clusters.

To visualize the characteristics of the four segments, PCA was conducted. Figure 5 illustrates elements of a PCA map, using data from Table A1, Table A2, Table A3, Table A4, Table A5 and Table A6 in Appendix A. It is important to note that this data was used in the creation of a singular PCA map, but the map was divided into four figures for ease of viewing and interpretation. In conjunction, the map explains 93.8% of the variability in the analysis conducted on this data, with 73.2% of the variability explained by dimension 1 (the horizontal axis) and 20.6% of the variability explained by dimension 2 (the vertical axis). Visually, this map summarizes the results through the location of points, grouped, indicating a high association. As the horizontal axis explains a large proportion of the variability in this data, associations were primarily determined by distances between points horizontally.

Jointly, the maps characterize Greenthusiasts as having an eco-conscious lifestyle with respect to environmental considerations (Figure 5a) and food purchasing factors (Figure 5d). They also exhibit very low neophobic tendencies (Figure 5b), high willingness to consume both sustainable and upcycled foods (Figure 5c), little to no hesitancy in purchasing or consuming upcycled foods, and a non-restrictive diet (Figure 5d). For the most part, Balanced Believers are comparable to Greenthusiasts. However, this segment is distinguished by a slightly healthier lifestyle (Figure 5a,d) and higher neophobic tendencies towards food technology (Figure 5b). Eco-Skeptics are characterized by their lifestyles, which reveal a strong social desire, with higher trust in strangers and the food industry (Figure 5a). They have restrictive diets and place high importance on packaging-related factors, such as brand, logo, or certification (Figure 5d). Additionally, they are very hesitant towards consuming both sustainable and upcycled foods (Figure 5c,d). Healthy Hesitants are similar to Eco-Skeptics but have a more health-focused lifestyle, distinguished by responses from the food lifestyle assessment (Figure 5a), diet, and food purchasing factors (Figure 5d). While the vertical axis explains a much smaller percentage of the variability compared to the horizontal axis, the location between points vertically is still critical in the interpretation of these maps. Primarily, the vertical axis indicates a complete distinction of Healthy Hesitants (in Quadrant I, upper right) from points located in Quadrant III (bottom left). This reaffirms their unwillingness and hesitancy to consume upcycled foods, as well as their more restrictive diet.

### 3.2. Segment Comparison

#### 3.2.1. Demographics

Listed in Table 2 are the percentages of participants within each cluster, corresponding to the demographic listed. The study population (*n* = 947) was primarily Generation Z (18–27, 39.4%) and Millennials (28–43, 29.9%), female (72.3%), White or Caucasian (68.6%), employed full-time (55.9%), and had a college degree (79.6%). The majority of respondents were from the South/South East (63.8%) or West (32.8%). Examining the clusters, Healthy Hesitants and Balanced Believers have the largest percentage of Generation Z (18–27) participants, while Eco-Skeptics and Greenthusiasts have the largest percentage of Millennials (28–43). Balanced Believers have the highest percentage of female respondents, followed by Greenthusiasts, Healthy Hesitants, and then Eco-Skeptics. Despite the population being primarily White or Caucasian, Healthy Hesitants, Balanced Believers, and Eco-Skeptics have a meaningful size of Asian or Pacific Islander respondents. Greenthusiasts have the highest percentage of respondents with a college education, followed by Balanced Believers, Healthy Hesitants, and Eco-Skeptics. Similar trends were observed for employment status. Greenthusiasts have the largest proportion of participants with a household income greater than USD 100,000 per year, comprising 38.6% of the group. Regarding regions of current residence, Balanced Believers have the highest percentage of participants from the South/South East, and Greenthusiasts have the highest percentage from the West and the lowest from the South/South East, while Eco-Skeptics have the lowest percentage of participants from the West. Eco-Skeptics appeared to have the highest percentage of respondents originating from the South/South East.

#### 3.2.2. Awareness and Familiarity

Table 3 shows the percentage of responses to statements of awareness regarding sustainable and upcycled foods between the clusters, using a 3-point Likert scale (No, I have never heard of it; Yes, I have heard of it but I don’t know what it means; Yes, I have heard of it and I know what it means). Average responses indicate the population has high awareness of sustainability, but low awareness of upcycling. Greenthusiasts showed the highest awareness of sustainability and upcycling, knowing what both terms mean. This is followed by Balanced Believers, Healthy Hesitants, and then Eco-Skeptics. Greenthusiasts also had the highest percentage of respondents who have heard of upcycled foods but do not know what they mean (33.3%). On the other hand, Eco-Skeptics have the highest percentage of participants who have heard of sustainable foods but do not know what they mean (50.0%). Healthy Hesitants reported the lowest awareness for both sustainable (7.7%) and upcycled (53.8%) foods, having never heard of either.

Familiarity with sustainable foods is shown in Table 4, while familiarity with upcycled foods is shown in Table 5. Participants’ numbers in this section were lower as familiarity with sustainable and upcycled foods was only collected for those who had selected either of the two “Yes” statements (Table 3) for their respective terms. Responses listed in Table 4 and Table 5 were recorded on a 3-point Likert scale (No, Maybe, Yes). All four clusters follow a similar trend, with higher overall familiarity with sustainable foods than with upcycled foods. Greenthusiasts primarily report the highest percentage of familiarity and Eco-Skeptics the lowest familiarity with the two types of foods. Aside from selecting that they are familiar with the concept of sustainability, the highest “Yes” response for all four groups is towards having heard about sustainable foods through marketing or word of mouth. The two statements with the lowest percentage of “Yes” responses included having purchased and having read or seen documentaries about sustainable foods. Balanced Believers and Greenthusiasts have a lower response towards reading or watching videos, while Eco-Skeptics and Healthy Hesitants have a lower response to purchasing. Despite having a much lower response to sustainability, all four groups report the highest percentages towards being familiar with the concept of upcycling. Greenthusiasts also have the highest percentage of respondents who know what upcycled food is (51.56%). Balanced Believers and Greenthusiasts have the lowest response to having purchased upcycled foods. Healthy Hesitants’ lowest response is towards purchasing and reading/watching documentaries about upcycled foods (both 20.83%). Eco-Skeptics’ lowest response is towards purchasing and eating upcycled foods (both 27.50%).

#### 3.2.3. Product Recognition and Role of Information

Table 6 shows the percentage of participants who correctly identified products as either sustainable or upcycled in each cluster pre- and post-infographic intervention, as well as the change in those percentages after being presented with infographics on sustainability and upcycling. Greenthusiasts had the highest initial recognition for nearly all products, aside from sustainable cookies and upcycled tomato, where Balanced Believers had a higher recognition. A general trend was observed of Greenthusiasts having the highest recognition pre- and post-intervention, which was followed by Balanced Believers, Healthy Hesitants, and then Eco-Skeptics. There was also a trend for higher initial recognition of sustainable products rather than upcycled products for all four segments. On average, there was an increase in participants being able to correctly identify the product type, with upcycled whey and tomatoes having the highest average change in percentage (12.1 and 10.1, respectively), while upcycled granola and sustainable greens have the lowest (2.7 and 2.4, respectively). All groups have a greater recognition for upcycled products after viewing the infographics. Healthy Hesitants have the largest overall change in participants who correctly identified a product, which was the upcycled whey at a 14% increase. Meanwhile, Eco-Skeptics had the largest decrease for the sustainable ice cream at 1.1%

#### 3.2.4. Cost

After identifying product images and viewing sustainability and upcycling infographics (Figure 3 and Figure 4), participants responded to questions regarding the cost of upcycled foods and reasons for hesitation towards consuming them. Table 7 indicates participants’ willingness to pay for upcycled foods across clusters by percentage of single selection. For the entire study population, the most selected option was “I would buy an upcycled food product if the price was similar to the traditional product.” This response was also the most selected for all four segments in descending order of Greenthusiasts (75.2%), Balanced Believers (67.7%), Healthy Hesitants (57.2%), and Eco-Skeptics (40.7%). The second-highest consideration observed overall was for purchasing upcycled foods if the price was slightly less than that of the traditional product. This trend was also observed for Eco-Skeptics (38.4%), Healthy Hesitants (30.3%), and Balanced Believers (20.5%). Greenthusiasts had the highest percentage for being willing to pay slightly more for an upcycled food product (15.7%). Only 2.3% of all respondents reported not being willing to purchase regardless of price. The majority of these participants belong to Eco-Skeptics, as 16.3% of this segment selected this response. No respondents from either Greenthusiasts or Balanced Believers reported not being willing to purchase regardless of price.

## 4. Discussion

### 4.1. Cluster Segmentation

Segmentation resulted in a total of four segments, with two (Greenthusiasts and Healthy Hesitants) being the most receptive to upcycled foods. Both of these segments display a higher agreement with environmentally conscious statements. This is consistent with the literature [7,9,19,38], where those who had higher environmental awareness and a more frugal perspective tend to be more accepting of upcycled foods. These two segments also exhibited lower food and food technology neophobia, which agrees with Aschemann-Witzel et al. [7], Coderoni and Perito [12], Hellali and Koraï [27], Perito et al. [28], and Tsimitri et al. [29], who suggested that neophobia had a negative relationship with upcycled food acceptability. Both segments revealed some technology neophobia, being uncertain about new technologies, switching too quickly, the long-term negative effects, and unbiased views in the media. With technology, research has shown an association between knowledge and positive attitudes [67] or lack of knowledge and negative attitudes [68]. Therefore, this result may be attributed to a lack of knowledge or awareness of the technologies used in food production. Eco-Skeptics were found to be status seeking, though this result did not concur with that found by McCarthy et al. [9], who concluded that status seeking positively influenced consumers’ willingness to purchase upcycled vegetable products.

Additionally, these two segments showed the highest agreement with consuming sustainable and upcycled foods when given descriptors of the two types. Healthy Hesitants appeared to be more willing to consume sustainable foods but not upcycled foods, which corresponds to some of their behavioral agreements as well, such as higher rated environmental responses, while having high neophobia, high hesitation to consume upcycled foods, and lower willingness to consume foods with upcycled food descriptors. This implies environmental consciousness, but, due to neophobia, they may be more reluctant to the new, unfamiliar concept of upcycled foods.

### 4.2. Segment Comparison

#### 4.2.1. Demographics

The purpose of comparing the clusters was to determine factors that indicate higher acceptance of upcycled foods. Lu et al. [5] suggested the ambiguity of demographic influence on acceptance, which was also observed in this study. Though there is not much distinction, Greenthusiasts and Balanced Believers contain a higher population of younger participants, which agrees with some previous studies, which have noted younger consumers displaying a higher affinity for upcycled foods [6,9,15]. Some prior research stated that males showed a higher liking towards upcycled foods [11,12], but others also found the opposite [6,7,9]. This study did not have a large male population, but Eco-Skeptics has the highest proportion of males within that cluster, which coincides with the latter conclusion. Distributions of White or Caucasian participants in this study disagree with those of Murillo et al. [13], where participants with this ethnic background were the least likely to favor upcycled foods. However, Greenthusiasts comprised the highest proportion of White or Caucasian participants, while Eco-Skeptics consisted of the least. Greenthusiasts and Balanced Believers contained much higher percentages of participants who have a bachelor’s degree or higher and were employed full-time. The majority of participants were from either the South/South East or West, which was likely attributed to the methods of survey distribution. It is reasonable to assume that most participants from the South/South East reside in North Carolina, while most participants from the West reside in California. Kennedy and Tyson [69] found that people living in the West were more likely to recognize the impacts of climate change compared to other regions of the US. This correlates to the findings in this study, as Greenthusiasts, the most environmentally conscious group, comprised the highest percentage of participants who currently reside in the West. Income revealed few differences between the segments.

#### 4.2.2. Diet and Purchasing of Food Factors

Diet and food purchasing factors help to reveal more behavioral attributes about consumers. Previous research on upcycled foods has not concluded the significant effects of dietary restrictions on acceptance. In this survey, however, Greenthusiasts appear to consume the least restrictive diet, while Eco-Skeptics have the most. This indicates that a lack of dietary restrictions leads to higher acceptance of upcycled foods. Greenthusiasts rated sustainability and social impact as more important purchasing factors compared to Eco-Skeptics, which aligns with the previous conclusion that Greenthusiasts are a more eco-conscious group [7,9,12,19,28,38]. On the other hand, Eco-Skeptics and Healthy Hesitants placed high importance on packaging-related factors, such as brand, label, and certification. This somewhat contradicts previous findings, as Coderoni and Perito [12,16] concluded higher purchase intentions from consumers who paid attention to product brands and labels but lower purchase intentions for certifications. The rest of the food purchasing factors did not reveal much differentiation between the segments, as the range between agreements was small.

#### 4.2.3. Awareness and Familiarity

Prior research has implied that through messaging and, therefore, awareness, consumer acceptance of upcycled foods increases [16,38,39,40]. Greenthusiasts and Balanced Believers displayed the highest percentage of participants who were aware of both sustainable and upcycled foods, supporting this idea of awareness aiding acceptability. There were a few participants who responded “No” to the familiarity category of questions due to the fact that only those who had heard of sustainable and upcycled foods were presented with those questions. Greenthusiasts and Balanced Believers reported higher percentages towards having heard about both sustainable and upcycled foods through marketing or word of mouth, as opposed to videos or documentaries. As current familiarity with upcycled foods remains low, relative to familiarity with sustainable foods, it is vital to increase marketing as that appears to be the main form of communication for consumers.

#### 4.2.4. Product Recognition and Role of Information

Generally, participants in all segments were able to differentiate upcycled foods from other forms of sustainable foods. All segments had higher recognition for sustainable products compared to upcycled products, likely due to more awareness of sustainability. Greenthusiasts had the highest recognition of upcycled products, also likely due to the large proportion of participants who were previously aware of the concept. After being shown infographics of sustainable and upcycled foods, nearly all segments improved their recognition. This suggests that messaging can aid in improving acceptance through increased awareness amongst consumers [16,38,39,40]. Although Greenthusiasts did not have as drastic a change in their recognition of upcycled products compared to the other segments, this result is not surprising, as their higher initial recognition confirms that many participants within this segment were already aware of the concept. The different product categories varied in their increase in recognition. No segments showed a decrease in upcycled food recognition, though the upcycled granola did have the lowest change. Greenthusiasts displayed a smaller change in recognition but higher initial recognition. The upcycled granola followed a similar trend, which may indicate that presenting information through infographics has a limited ability to educate consumers. Further investigation into these product categories and their acceptance when presented with information is needed.

#### 4.2.5. Cost and Hesitation

After learning about upcycled foods, Greenthusiasts and Balanced Believers appeared to be the most willing to purchase. It is unknown what effect informing participants of sustainable and upcycled foods had on these responses, but these results agree with previous indicators of the two segments being the most willing to purchase [6,7,9,16,19,38,39,40]. The cost question also revealed that most participants would want upcycled foods to be priced similarly or less than their conventional counterparts. Previous studies have also found price sensitivity to be a point of concern for consumers [8,31,32,34]. Healthy Hesitants and Eco-Skeptics were the most reluctant to purchase and the most hesitant towards consuming upcycled foods. Future research should explore in-depth educational messaging to Healthy Hesitants and Eco-Skeptics to determine if they could become more receptive.

### 4.3. Limitations and Future Work

The design of the project was similar to that of Rovai et al. [41], and the limitations of this study are comparable. Due to distribution methods, participants were primarily from the South/South East and the West. Though the majority of participants came from the South/South East, there was still a large percentage of participants from the West (likely California). As California may be perceived as more environmentally conscious and healthier than other regions of the U.S., it may have skewed the results towards higher populations in the more accepting clusters. Another demographic limitation was the gender distribution. A large majority of the survey population was women, who have been found to be more health- and environmentally conscious, also having a higher willingness to purchase or try upcycled foods, while men have been found to be more skeptical of upcycled foods [6,7,9]. Since there is roughly an even split between the two genders in the U.S., this study was not representative of the overall U.S. population. Additionally, the databases in California and North Carolina, where this survey was distributed, often conduct sensory testing with various products, potentially pre-exposing participants from this study to novel food products, thus increasing their openness to a novel food category, such as upcycled foods. Due to the survey methodology, this study was conducted entirely online. Therefore, respondents included in this study were limited to those who had internet and digital access, with a reliance on the two sensory databases and researcher connections through social media.

The conceptual nature of the survey is also another limitation. Questions regarding acceptance, purchase intent, and consumption were asked strictly with imagery of products as a reference. The sensory attributes of food play a vital role in its acceptance. Therefore, without a physical product for consumers to evaluate, their acceptance of upcycled foods cannot be determined. There have already been many studies testing the incorporation of upcycled ingredients in new products. However, future work should conduct sensory testing with consumer segments to identify the true acceptability of upcycled foods, validating the results of this study.

Another limitation was the products on the market. The product recognition portion of this study did not contain matching or similar products between sustainable and upcycled foods for all five categories, as described by Bhatt et al. [30]. This may be due to the lack of upcycled products on the market, especially those without an upcycled label on the front panel of their packaging. Additionally, by finding solutions to various waste streams of food, developers have created novel upcycled food products, such as chicken skin crisps [47]. As more upcycled products appear on the market, research should explore how they compare to both their sustainable and conventional counterparts, whether that is evaluating recognition or acceptance.

## 5. Conclusions

This study identified consumer segments for upcycled foods, which, when their attributes are considered, can provide actionable insights for targeted marketing and product development. Among the four groups, Greenthusiasts and Balanced Believers emerged as the most receptive. Greenthusiasts, defined by strong environmental consciousness, low food neophobia, high familiarity with upcycled foods, and flexible diets, show little hesitation in purchasing these products. Balanced Believers, while somewhat more cautious, share similar flexibility in their diets and consider environmental impacts when making purchases. Though Greenthusiasts are slightly more accepting, the differences between the two groups may be less meaningful from an industry perspective, given their shared openness and low resistance to food technologies. Marketing efforts aimed at both groups should, therefore, emphasize the environmental benefits of upcycled foods. From a product development standpoint, their dietary flexibility suggests they would be willing to engage with a variety of upcycled ingredients, including those derived from gluten, dairy, or high-fat sources, broadening innovation opportunities.

In contrast, Healthy Hesitants and Eco-Skeptics expressed greater reluctance. Healthy Hesitants, despite valuing health-oriented choices, show elevated food and technology neophobia, resulting in hesitancy toward upcycled foods. Eco-Skeptics represent the most resistant segment, marked by distrust, resistance to change, and the highest levels of neophobia. This group also demonstrates more dietary restrictions, further limiting openness to new food products. Overall, food and technology neophobia emerged as significant barriers to adoption, while fewer dietary restrictions correlated with higher acceptance. This suggests that flexibility in dietary practices plays a key role in these groups’ consumer receptiveness.

Price expectations also play a critical role in market adoption by consumers of these products. Consumers generally prefer upcycled foods to be priced at or below the cost of conventional alternatives. This preference presents a potential opportunity for producers, as upcycled ingredients may have reduced manufacturing costs. If realized, these savings could allow producers to price upcycled products competitively, potentially even below traditional products, thereby strengthening consumer appeal.

Future research should continue exploring the consumers’ sensory evaluation of both sustainable and upcycled foods to understand how consumers evaluate such products. In addition, future work should consider consumer willingness to pay for specific upcycled food attributes, particularly when production efficiencies also create cost advantages. Understanding the intersection of consumer evaluation and acceptance, neophobia, dietary habits, and price sensitivity will be essential for guiding industry strategies. Ultimately, these findings highlight both the promise and complexity of positioning upcycled foods for broader adoption.

## Figures and Tables

**Table 1:** List of products used for sustainable and upcycled product recognition.

Product Name	Product Type	Brand	Image Source	Brand Link
Whey Protein Blend	Upcycled	Ascent	https://ascentprotein.com (accessed on 19 July 2025) [52]	https://ascentprotein.com (accessed on 19 July 2025) [52]
Cookies	Upcycled	Fancypants	https://us.amazon.com/Fancypants-Cookies-Buttery-Delicious-Choco-%20862%20late/dp/B07RZMRNRD (accessed on 22 July 2025) [48]	https://fancypantsbakery.com (accessed on 20 January 2025) [49]
Granola Bar	Upcycled	Regrained	www.foodgal.com/2016/03/dont-just-drink-beer-eat-it-too/ (accessed on 19 July 2025) [50]	https://upcycledfoods.com/ingredients/ (accessed on 19 July 2025) [51]
Chicken Skin Crisps	Upcycled	Flock Foods	https://boxncase.com/products/flock-chicken-skin-crisps-original-2-5-oz-bag (accessed on 19 July 2025) [46]	https://flockfoods.com (accessed on 19 July 2025) [47]
Tomato Sauce	Upcycled	Matriark Foods	https://thrillist.com/eat/nation/matriark-foods-climate-friendly-pantry-staples (accessed on 19 July 2025) [53]	https://matriarkfoods.com (accessed on 19 July 2025) [54]
Avocados and Bananas	Neither (Attention Check)	N/A	https://storymoneyimpact.com/resources-interceptors/ (accessed on 19 July 2025) [63]	https://storymoneyimpact.com/resources-interceptors/ (accessed on 19 July 2025) [63]
Plant-based Beef	Sustainable	Impossible Foods	https://impossiblefoods.com (accessed on 19 July 2025) [55]	https://impossiblefoods.com (accessed on 19 July 2025) [55]
Lettuce	Sustainable	Revol Greens	https://bluebookservices.com/revol-greens-releases-three-new-products/ (accessed on 22 July 2025) [62]	https://revolgreens.com (accessed on 19 July 2025) [61]
Cookies	Sustainable	Rule Breaker	https://target.com/p/rule-breaker-singles-variety-pack-4-1-9-oz/-/A-93108275 (accessed on 22 July 2025) [57]	https://rulebreakersnacks.com (accessed on 19 July 2025) [56]
Cereal	Sustainable	Grain Berry	https://grainberry.com (accessed on 19 July 2025) [58]	https://grainberry.com (accessed on 19 July 2025) [58]
Ice Cream	Sustainable	Brave Robot	https://techcrunch.com/2020/07/15/brave-robot-ice-cream-launches-as-the-first-brand-from-the-perfect-day-backed-urgent-company/ (accessed on 19 July 2025) [60]	https://perfectday.com/blog/brave-robots-animal-free-ice-cream-the-future-favors-the-brave/ (accessed on 19 July 2025) [59]

**Table 2:** Demographics summary.

Demographics	Average *n* = 947	Greenthusiasts *n* = 306	Balanced Believers *n* = 347	Healthy Hesitants *n* = 208	Eco-Skeptics *n* = 86
Age					
18–27	39.4	36.6	41.8	44.2	27.9
28–43	29.9	34.6	30.0	20.2	36.0
44–59	19.0	23.2	16.1	17.3	19.8
60–78	11.3	5.2	12.1	17.3	15.1
79–96	0.4	0.3	0.0	1.0	1.2
97 and Over	0.0	0.0	0.0	0.0	0.0
Gender					
Male	26.0	23.5	25.1	26.9	36.0
Female	72.3	73.2	74.1	72.1	62.8
Not Listed	0.8	2.0	0.3	0.5	0.0
Prefer Not to Answer	0.8	1.3	0.6	0.5	1.2
Ethnic Background					
White or Caucasian	68.6	79.1	69.2	57.2	57.0
Hispanic or Latino	10.3	12.7	8.6	10.6	8.1
Black or African-American	5.3	2.3	4.6	7.2	14.0
Native American or American Indian	0.8	0.7	0.6	1.0	2.3
Asian or Pacific Islander	20.9	12.7	22.8	29.3	22.1
Not Listed	0.8	1.0	1.2	0.5	0.0
Prefer Not to Answer	1.2	1.3	1.2	1.4	0.0
Education Level					
Some High School	0.0	0.0	0.0	0.0	0.0
High School Graduate or Equivalent	4.5	2.6	3.7	8.2	5.8
Some College	14.6	13.1	15.3	14.9	16.3
Trade, Technical or Vocational Training	1.4	1.6	0.9	1.9	1.2
Associate Degree	5.8	4.2	6.6	5.3	9.3
Bachelor’s Degree	40.7	42.5	45.0	36.1	27.9
Master’s Degree	26.8	27.1	24.2	28.4	32.6
Professional Degree	1.1	2.0	0.3	1.4	0.0
Doctorate Degree	5.2	6.9	4.0	3.8	7.0
Student Status					
Yes	36.7	32.0	39.2	40.4	34.9
No	63.3	68.0	60.8	59.6	65.1
Employment Status					
Not Employed	11.0	8.2	11.8	12.5	14.0
Employed Part-Time	20.5	17.3	20.5	25.5	19.8
Employed Full-Time	55.9	67.3	54.8	45.2	45.3
Self Employed	2.4	2.0	2.6	2.4	3.5
Retired	6.7	3.6	6.1	10.1	11.6
Full-time Homemaker	3.6	1.6	4.3	4.3	5.8
Household Income					
Under USD 20,000	10.7	7.8	10.1	15.9	10.5
USD 20,000 to USD 49,999	16.9	15.0	18.2	18.3	15.1
USD 50,000 to USD 74,999	18.2	17.3	18.4	16.8	23.3
USD 75,000 to USD 99,999	14.5	15.4	15.6	12.5	11.6
USD 100,000 to USD 149,999	18.3	23.2	16.7	14.4	16.3
USD 150,000 or more	14.5	15.4	13.8	14.4	14.0
Prefer not to answer	7.1	5.9	7.2	7.7	9.3
Current Residence					
West	32.8	39.2	30.8	29.3	26.7
Midwest	1.8	1.6	1.2	2.4	3.5
South/South East	63.8	58.2	67.4	64.9	66.3
North East	1.6	1.0	0.6	3.4	3.5
Outside of U.S.	0.0	0.0	0.0	0.0	0.0
Origin					
West	31.9	36.9	31.1	29.3	23.3
Midwest	8.3	9.5	7.2	8.7	8.1
South/South East	35.4	32.4	38.3	32.2	41.9
North East	10.5	13.1	9.5	7.7	11.6
Outside of U.S.	13.9	8.2	13.8	22.1	15.1

**Table 3:** Percent responses to sustainability and upcycling awareness by cluster.

Awareness	Average *n* = 947	Greenthusiasts *n* = 306	Balanced Believers *n* = 347	Healthy Hesitants *n* = 208	Eco-Skeptics *n* = 86
Sustainability					
No, I have never heard of it	4.3	2.6	3.2	7.7	7.0
Yes, I have heard of it but I don’t know what it means	37.8	32.4	35.7	44.2	50.0
Yes, I have heard of it and I know what it means	57.9	65.0	61.1	48.1	43.0
Upcycling					
No, I have never heard of it	44.4	37.3	42.7	53.8	53.5
Yes, I have heard of it but I don’t know what it means	30.8	33.3	32.9	26.0	25.6
Yes, I have heard of it and I know what it means	24.8	29.4	24.5	20.2	20.9

**Table 4:** Percent responses to statements of sustainable food familiarity of participants who selected either “Yes”, “No”, or “Maybe” option for sustainability awareness (Greenthusiasts *n* = 298; Balanced Believers *n* = 336; Healthy Hesitants *n* = 192; Eco-Skeptics *n* = 80).

Familiarity of Sustainable Food	Average *n* = 906	Greenthusiasts *n* = 298	Balanced Believers *n* = 336	Healthy Hesitants *n* = 192	Eco-Skeptics *n* = 80
I am familiar with the concept of sustainability					
No	3.31	2.01	2.38	4.17	10.00
Maybe	15.34	6.04	13.39	25.00	35.00
Yes	81.35	91.95	84.23	70.83	55.00
I know what sustainable food is					
No	9.60	6.71	10.12	8.85	20.00
Maybe	30.46	27.18	28.57	36.46	36.25
Yes	59.93	66.11	61.31	54.69	43.75
I can think of examples of sustainable food(s)					
No	18.87	13.09	19.94	20.31	32.50
Maybe	30.13	26.85	30.36	34.38	31.25
Yes	50.99	60.07	49.70	45.31	36.25
I have eaten sustainable food(s)					
No	5.52	2.01	5.65	5.21	18.75
Maybe	40.40	32.21	41.67	47.92	47.50
Yes	54.08	65.77	52.68	46.88	33.75
I have purchased sustainable food(s)					
No	9.38	4.36	10.42	8.85	25.00
Maybe	42.27	36.91	43.15	48.96	42.50
Yes	48.34	58.72	46.43	42.19	32.50
I have read about and/or seen videos/documentaries about sustainable food(s)					
No	27.59	23.83	28.27	29.17	35.00
Maybe	26.60	22.48	29.76	27.60	26.25
Yes	45.81	53.69	41.96	43.23	38.75
I have heard about sustainable food(s) through marketing (products) and/or word of mouth					
No	10.60	8.05	12.50	8.85	16.25
Maybe	26.16	22.15	25.89	28.13	37.50
Yes	63.25	69.80	61.61	63.02	46.25

**Table 5:** Percent responses to statements of upcycled food familiarity of participants who selected either “Yes”, “No”, or “Maybe” option for upcycling awareness (Greenthusiasts *n* = 192; Balanced Believers *n* = 199; Healthy Hesitants *n* = 96; Eco-Skeptics *n* = 40).

Familiarity of Upcycled Food	Average *n* = 527	Greenthusiasts *n* = 192	Balanced Believers *n* = 199	Healthy Hesitants *n* = 96	Eco-Skeptics *n* = 40
I am familiar with the concept of upcycling					
No	11.39	6.77	11.56	17.71	17.50
Maybe	28.08	23.44	32.16	26.04	35.00
Yes	60.53	69.79	56.28	56.25	47.50
I know what upcycled food is					
No	14.99	13.02	18.09	13.54	12.50
Maybe	39.66	35.42	40.20	43.75	47.50
Yes	45.35	51.56	41.71	42.71	40.00
I can think of examples of upcycled food(s)					
No	30.36	27.60	32.66	30.21	32.50
Maybe	33.59	30.21	35.18	35.42	37.50
Yes	36.05	42.19	32.16	34.38	30.00
I have eaten upcycled food(s)					
No	17.65	11.98	16.08	23.96	37.50
Maybe	53.89	55.21	57.79	51.04	35.00
Yes	28.46	32.81	26.13	25.00	27.50
I have purchased upcycled food(s)					
No	29.22	20.83	32.16	33.33	45.00
Maybe	47.25	51.04	48.24	45.83	27.50
Yes	23.53	28.13	19.60	20.83	27.50
I have read about and/or seen videos/documentaries about upcycled food(s)					
No	43.07	41.15	48.24	41.67	30.00
Maybe	26.94	24.48	23.62	37.50	30.00
Yes	29.98	34.38	28.14	20.83	40.00
I have heard about upcycled food(s) through marketing (products) and/or word of mouth					
No	24.10	21.35	28.64	22.92	17.50
Maybe	32.45	32.29	29.15	37.50	37.50
Yes	43.45	46.35	42.21	39.58	45.00

**Table 6:** Percentage of correctly identified products (as sustainable or upcycled) pre- and post-infographic intervention and change (Δ) in percentage of correctly identified products after intervention with sustainability and upcycling infographics.

Product Concepts	Greenthusiasts *n* = 306			Balanced Believers *n* = 347			Healthy Hesitants *n* = 208			Eco-Skeptics *n* = 86			Average *n* = 947
	Pre	Post	Δ	Pre	Post	Δ	Pre	Post	Δ	Pre	Post	Δ	Δ
Sustainable													
Beef	93.5	97.1	3.6	90.2	94.2	4	85.6	90.4	4.8	79.1	79.1	0.0	3.1
Greens	97.7	97.4	−0.3	91.9	95.1	3.2	89.9	89.4	−0.5	81.4	88.4	7.0	2.4
Cookies	90.8	95.4	4.6	91.1	94.8	3.7	87.5	91.3	3.8	81.4	84.9	3.5	3.9
Cereal	88.6	92.8	4.2	82.1	90.2	8.1	77.9	85.6	7.7	79.1	83.7	4.6	6.2
Ice Cream	91.2	96.4	5.2	88.2	92.5	4.3	83.2	89.4	6.2	80.2	79.1	−1.1	3.7
Upcycled													
Whey	83.3	94.8	11.5	79.0	90.2	11.2	71.6	85.6	14	69.8	81.4	11.6	12.1
Cookies	92.2	97.4	5.2	89.6	95.7	6.1	83.2	90.4	7.2	74.4	81.4	7.0	6.4
Granola	97.7	98.4	0.7	96.0	96.8	0.8	89.4	94.2	4.8	84.9	89.5	4.6	2.7
Chicken	93.5	97.4	3.9	89.6	96.3	6.7	86.5	91.8	5.3	75.6	83.7	8.1	6.0
Tomato	81.0	92.2	11.2	81.3	89.6	8.3	76.9	86.1	9.2	74.4	86.0	11.6	10.1

**Table 7:** Percent willingness to pay for upcycled foods by cluster.

Cost	Average *n* = 947	Greenthusiasts *n* = 306	Balanced Believers *n* = 347	Healthy Hesitants *n* = 208	Eco-Skeptics *n* = 86
I would be willing to pay slightly more for an upcycled food product	11.7	15.7	11.8	8.7	4.7
I would buy an upcycled food product if the price was similar to the traditional product	65.4	75.2	67.7	57.2	40.7
I would buy an upcycled food product if the price was slightly less than the traditional product	20.6	9.2	20.5	30.3	38.4
I would NOT buy an upcycled food product, regardless of price	2.3	0.0	0.0	3.8	16.3

## Data Availability

The data presented in this study are available upon request from the corresponding author. The data are not publicly available due to privacy and ethical restrictions.

## Supplementary Materials

The following supporting information can be downloaded at https://www.mdpi.com/article/10.3390/foods14203508/s1, Document S1: Sustainable and upcycled food survey.

## Abbreviations

The following abbreviations are used in this manuscript:

U.S.	United States
FNS	Food Neophobia Scale
FTNS	Food Technology Neophobia Scale
CATA	Check-all-that-apply
PCA	Principal Component Analysis

## Appendix A

**Table A1:** Mean agreement of Food Lifestyle Assessment (FLA) and Basic Lifestyle Assessment (BLA) statements (on a 5-point Likert scale, 1 = Strongly Disagree to 5 = Strongly Agree).

FLA and BLA Statements	Average *n* = 947	Greenthusiasts *n* = 306	Balanced Believers *n* = 347	Healthy Hesitants *n* = 208	Eco-Skeptics *n* = 86
FLA: I am willing to pay more for all natural or organic foods	3.4	3.4	3.3	3.4	3.3
FLA: I am concerned about the environmental impact of the foods I eat	3.7	4.0	3.6	3.6	3.2
FLA: It’s difficult for me to understand what products are truly nutritious and/or healthy for me and my family	2.7	2.5	2.8	2.9	3.2
FLA: I am willing to modify my diet in order to be healthier	4.2	4.3	4.2	4.2	3.9
FLA: I am concerned about the food I consume going to waste	4.1	4.3	4.0	4.0	3.5
FLA: I am always looking for new foods to help me live a healthier life	3.8	3.8	3.9	3.8	3.6
FLA: I and/or other members of my household have food allergies that prevent us from eating certain foods	2.1	1.8	2.2	2.1	3.0
FLA: I believe organic food is more sustainable	3.3	3.2	3.2	3.5	3.4
FLA: I am able to trust the food industry	2.8	2.6	2.8	2.6	3.2
FLA: I think about examples and benefits of food products	3.8	3.9	3.8	3.6	3.5
FLA: I crave sweet foods	4.0	4.0	3.9	4.0	3.9
FLA: I use food as a reward	3.4	3.3	3.4	3.5	3.3
FLA: I buy new types of food earlier than other people	3.0	3.0	3.0	2.8	2.9
BLA: I am loyal to brands that I like	3.8	3.7	3.8	3.9	3.9
BLA: I exercise regularly	3.7	3.7	3.7	3.6	3.7
BLA: I am able to trust most strangers	2.6	2.7	2.6	2.3	2.8
BLA: It is important that others like the products and brands I buy	2.1	1.7	2.1	2.2	2.9
BLA: Sometimes I buy a product because my friends do so	3.0	2.9	3.1	3.0	3.0
BLA: Name-brand purchase is a good way to distinguish people from others	2.1	1.8	2.1	2.3	3.0
BLA: Name products and brands purchased can bring me a sense of prestige	2.3	1.9	2.4	2.6	3.0
BLA: I purchase certain products because it is morally the right thing	3.0	3.2	3.0	2.9	3.1
BLA: I often try to avoid products that are bought by the general population	2.2	2.0	2.2	2.4	2.8
BLA: I would never throw away things that are still useful	3.9	4.0	3.8	3.8	3.6
BLA: I only buy what I need	3.3	3.2	3.2	3.4	3.6
BLA: If I can re-use an item I already have, there’s no sense in buying something new	4.2	4.3	4.1	4.1	3.8

**Table A2:** Mean agreement of Food Neophobia Scale (FNS) and Food Technology Neophobia Scale (FTNS) statements (on a 7-point scale, 1 = Strongly Disagree to 7 = Strongly Agree).

FNS and FTNS	Average *n* = 947	Greenthusiasts *n* = 306	Balanced Believers *n* = 347	Healthy Hesitants *n* = 208	Eco-Skeptics *n* = 86
FNS: I am constantly sampling new and different foods	5.0	5.5	5.1	4.6	4.3
FNS: I don’t trust new foods	2.8	2.1	2.7	3.4	4.2
FNS: If I don’t know what is in a food, I won’t try it	3.7	2.8	3.7	4.6	4.9
FNS: I like foods from different countries	6.0	6.5	6.1	5.8	4.5
FNS: Ethnic food looks too weird to eat	2.0	1.3	1.9	2.5	4.1
FNS: At dinner parties, I will try a new food	5.8	6.4	6.0	5.3	4.6
FNS: I am afraid to eat things I have never had before	2.7	1.8	2.6	3.6	4.4
FNS: I am very particular about the foods I will eat	3.4	2.4	3.2	4.6	4.9
FNS: I will eat almost anything	4.7	5.6	4.7	3.6	3.9
FNS: I like to try new ethnic restaurants	5.7	6.4	5.9	5.0	3.9
FTNS: There are plenty of tasty foods around so we don’t need to use new food technologies to produce more	3.2	2.8	3.0	3.5	4.6
FTNS: The benefits of new food technologies are often grossly overstated	3.7	3.5	3.5	4.2	4.4
FTNS: New food technologies decrease the natural quality of food	3.7	3.3	3.4	4.3	4.4
FTNS: There is no sense trying out high-tech food products because the ones I eat are already good enough	3.3	2.9	3.2	3.8	4.3
FTNS: New foods are not healthier than traditional foods	3.7	3.5	3.6	4.0	4.1
FTNS: New food technologies are something I am uncertain about	4.2	3.9	4.1	4.7	4.6
FTNS: Society should not depend heavily on technologies to solve its food problems	3.6	3.2	3.5	4.3	4.5
FTNS: New food technologies may have long term negative environmental effects	4.3	4.1	4.1	4.7	4.4
FTNS: It can be risky to switch to new food technologies too quickly	4.6	4.4	4.4	4.9	4.7
FTNS: New food technologies are unlikely to have long term negative health effects	3.7	3.7	3.7	3.5	3.9
FTNS: New products produced using new food technologies can help people have a balanced diet	4.7	4.9	4.8	4.4	4.4
FTNS: New food technologies give people more control over their food choices	4.7	4.8	4.7	4.4	4.5
FTNS: The media usually provides a balanced and unbiased view of new food technologies	2.8	2.6	2.8	2.9	3.7

**Table A3:** Mean agreement of self-beliefs (SBCs) and beliefs of others (BOCs) regarding consuming sustainable and upcycled foods (on a 5-point Likert scale, 1 = Strongly Disagree to 5 = Strongly Agree).

SBC and BOC Statements	Average *n* = 947	Greenthusiasts *n* = 306	Balanced Believers *n* = 347	Healthy Hesitants *n* = 208	Eco-Skeptics *n* = 86
SBC: I would consume a plant-based product	4.4	4.8	4.4	4.0	3.4
SBC: I would consume a product that uses less water to be produced	4.3	4.8	4.4	3.9	3.4
SBC: I would consume a product that uses less energy to be produced	4.3	4.8	4.4	4.0	3.5
SBC: I would consume a product that uses less land to be produced	4.3	4.7	4.3	3.9	3.4
SBC: I would consume a product that rescues food from being wasted	4.1	4.6	4.2	3.6	3.3
SBC: I would consume a product that contains ingredient(s) that are not regarded or commonly seen as edible	2.8	3.3	2.8	2.3	2.4
SBC: I would consume a product that contains damaged or imperfect produce	3.8	4.5	3.9	3.1	2.9
SBC: I would consume a product that contains byproducts and/or scraps from other food preparation	3.5	4.1	3.5	2.8	2.8
BOC: I would support my family members or children eating sustainable foods	4.5	4.8	4.5	4.2	3.7
BOC: I think more people should consume sustainable foods	4.3	4.6	4.3	4.0	3.5
BOC: I think more people should consume upcycled foods	3.9	4.4	3.9	3.4	3.2
BOC: I would support my family members or children eating upcycled foods	4.2	4.6	4.2	3.7	3.3

**Table A4:** Percentage of individuals who responded to check-all-that-apply (CATA) statements about dietary restrictions.

Dietary Restrictions	Average *n* = 947	Greenthusiasts *n* = 306	Balanced Believers *n* = 347	Healthy Hesitants *n* = 208	Eco-Skeptics *n* = 86
Eat almost anything	56.1	70.3	53.3	45.7	41.9
Healthy eating, but no particular nutritional restrictions	57.6	58.5	59.9	54.3	52.3
On again, off again healthy eating	35.6	34.3	35.4	34.6	43.0
Flexitarian—primarily plant based with occasional meat	10.6	13.7	10.7	8.7	3.5
Clean—try to eat gluten free, soy-free, and organic when possible	6.5	5.2	7.8	5.3	9.3
Ketogenic	2.0	1.0	2.6	2.4	2.3
Paleo	1.8	1.6	1.4	2.4	2.3
Whole 30/Anti-inflammatory	3.8	2.6	3.7	3.4	9.3
Pescatarian—primarily vegetarian, eats fish and seafood	5.3	4.2	6.3	5.3	4.7
Vegetarian	9.7	8.5	8.6	14.4	7.0
Vegan (no animal products)	2.6	1.3	3.7	2.9	2.3
Gluten-free	4.6	2.3	6.9	3.8	5.8
Dairy-free	5.6	3.9	7.8	4.3	5.8
Intermittent fasting	13.4	11.4	13.8	14.9	15.1
Low-glycemic (e.g. low carb)	6.5	5.6	4.9	10.1	8.1
Other	3.5	2.0	4.0	4.8	3.5

**Table A5:** Mean ratings of importance corresponding to purchasing of food (POF) factors (on a 5-point Likert scale, 1 = Not important at all to 5 = Very important).

Purchasing of Food Factors	Average *n* = 947	Greenthusiasts *n* = 306	Balanced Believers *n* = 347	Healthy Hesitants *n* = 208	Eco-Skeptics *n* = 86
Cost	4.47	4.55	4.49	4.42	4.20
Convenience	4.01	3.95	4.03	4.05	4.01
Nutritional Value	4.44	4.49	4.46	4.42	4.24
Upcycled Content	2.91	2.99	2.87	2.89	2.83
Origin	3.34	3.24	3.30	3.51	3.43
Brand	2.78	2.36	2.81	3.10	3.34
Label	2.90	2.50	2.95	3.26	3.24
Logo	2.13	1.77	2.14	2.47	2.50
Certification	3.29	3.01	3.28	3.59	3.63
Taste	4.77	4.81	4.78	4.75	4.62
Quality	4.63	4.59	4.65	4.67	4.58
Product Category	3.41	3.29	3.47	3.48	3.48
Locality	3.52	3.55	3.51	3.51	3.43
Sustainability	3.58	3.78	3.48	3.59	3.28
Social Impact	2.97	3.14	2.86	3.02	2.67
Traceability	3.10	3.01	3.12	3.16	3.17

**Table A6:** Percentage of individuals who responded to CATA options about hesitations to purchase and/or consume upcycled foods.

Hesitation to Purchase or Consume Upcycled Food	Average *n* = 947	Greenthusiasts *n* = 306	Balanced Believers *n* = 347	Healthy Hesitants *n* = 208	Eco-Skeptics *n* = 86
I think that upcycled foods are disgusting	2.3	0.0	0.9	4.8	10.5
I have a feeling that upcycled foods would not taste good	23.1	11.1	19.6	33.7	54.7
I think that upcycled foods seem strange/unfamiliar	20.1	7.5	17.6	35.1	38.4
I think that upcycled foods would be more expensive than conventional foods	46.7	45.8	48.1	47.1	43.0
They are waste products and I would not like to have them in new foods	6.3	1.3	2.9	15.9	15.1
I am not interested in their health benefits	2.4	0.7	2.0	2.4	10.5
I am not interested in their environmental benefits	2.7	0.3	1.7	3.4	14.0
I am NOT hesitant to eat upcycled foods	51.2	68.6	55.0	33.2	17.4
Other	4.2	4.6	3.2	3.8	8.1
