# Supplementary material for: Upcycled vs. Sustainable: Identifying Consumer Segments and Recognition of Sustainable and Upcycled Foods Within the United States
Source: Foods. 2025 Oct 15;14(20):3508. doi: 10.3390/foods14203508 (PMC12562476; doi:10.3390/foods14203508)
Supplement: Supplementary file 1 [file foods-14-03508-s001.zip › foods-3848123-S1 - Preliminary test screener and demographics.pdf]

Survey Created Using RedJade Software

Questionnaire Page

1 **Which of the following best describes your age?**

- ○ Under 18
- ○ 18 – 27
- ○ 28 – 43
- ○ 44 – 59
- ○ 60 – 78
- ○ 79 – 96
- ○ 97 and Over

2 Please specify your gender.

- ◯ Male
- ◯ Female
- ◯ Not Listed
- ◯ Prefer Not to Answer

3 Are you currently pregnant or nursing?

- ◯ Yes
- ◯ No

4 Which of the following best describes your ethnic background? (Check all that apply)

- ☐ White or Caucasian
- ☐ Hispanic or Latino
- ☐ Black or African-American
- ☐ Native American or American Indian
- ☐ Asian or Pacific Islander
- ☐ Not Listed
- ☐ Prefer Not to Answer

5 What level of education have you completed?

- ◯ Some High School
- ◯ High School Graduate or Equivalent
- ◯ Some College
- ◯ Trade, Technical or Vocational Training
- ◯ Associate Degree
- ◯ Bachelor’s Degree
- ◯ Master’s Degree
- ◯ Professional Degree
- ◯ Doctorate Degree

6 Are you currently a student?

- ◯ Yes
- ◯ No

7 Which of the following best describes your employment status?

- ◯ Not Employed
- ◯ Employed Part-Time
- ◯ Employed Full-Time
- ◯ Self Employed
- ◯ Retired
- ◯ Full-time Homemaker

8 Which of the following best describes your annual household income?

- Under $20,000
- $20,000 to $49,999
- $50,000 to $74,999
- $75,000 to $99,999
- $100,000 to $149,999
- $150,000 or more
- Prefer not to answer

9 Please indicate what you are allergic or sensitive to. (Check all that apply)

- ☐ Peanut
- ☐ Treenut
- ☐ Milk/Dairy
- ☐ Egg
- ☐ Wheat/Gluten
- ☐ Soy
- ☐ Seafood/Fish
- ☐ Shellfish
- ☐ Sesame
- ☐ Other
- ☐ None (I do not have any allergies or food sensitivities)

10 Please indicate what you are **NOT** willing to eat. (Check all that apply)

- ☐ Oatmilk Flour
- ☐ Sunflower Protein Flour
- ☐ Date Syrup
- ☐ Millet Flour
- ☐ Flaxseed Meal
- ☐ Chia Seeds
- ☐ Spinach
- ☐ Cocoa
- ☐ Oat Hull Fibre
- ☐ Carrots
- ☐ Broccoli
- ☐ Tomatoes
- ☐ Beets
- ☐ Shiitake Mushrooms
- ☐ None of the Above (I am willing to eat all of the above)

11 **Which, if any, of the following products have you purchased and eaten in the past 3 months? (Check all that apply)**

- ☐ Bakery section items, such as brownies, cookies, or donuts
- ☐ Deli meat or cheese
- ☐ Fresh berries, such as strawberries, blackberries, blueberries, or raspberries
- ☐ Frozen appetizers, such as pizza rolls, egg rolls, or mozzarella sticks
- ☐ Ice cream, gelato, sorbet, or frozen yogurt
- ☐ Snack crackers, such as Wheat Thins, Triscuits, or Ritz
- ☐ None of the above

NOTE: Only answer this question if on question #1 of questionnaire page your answer was one of the following: "Bakery section items, such as brownies, cookies, or donuts"

12 Which, if any, of the following bakery products have you purchased and eaten in the past 3 months? (Check all that apply)

- ☐ Brownies
- ☐ Cookies
- ☐ Donuts
- ☐ Cake
- ☐ Bread
- ☐ Muffins

13 Have you had any colds, allergies, or sicknesses in the past two weeks?

- ◯ Yes
- ◯ No
- ◯ Don’t Know

14 Are you currently experiencing a lost of taste and/or smell?

- ◯ Yes
- ◯ No
- ◯ Don’t Know
